# The Earliest Herbals

**Published:** 1894-09-01

**Authors:** 


### The First Century of English Medical Literature.

### VIII.—THE EARLIEST HERBALS.

By far the most respectable of ancient medicines were those compounded from herbs. To a simple reliance on their use may be ascribed the great reputations which were often gained in country places by the "wise man" or "wise woman" whose cures far outdid those of the regular physician. The restless ingenuity of the learned doctor disdained the known qualities of the herbs with which every child was familiar, and lent itself to discovering the occult properties of substances which Macbeth's witches might have rejected with disgust. But the herbs held their own in popular esteem, and even among some of the learned, in spite of being hardly pushed by the new chemical remedies which towards the close of the sixteenth century were fully in vogue.

A curious little pamphlet by "T. B.," printed in 1580, complains of "the unmeasurable cost these strange medicines put us to." From this it appears that medicine to cure a patient's rheum would sometimes amount to half his substance, while the curing of a palsy or colic would absorb what might have kept a whole household in comfort for a year. "T. B." is concerned not merely for the cost, but also on account of the danger of these foreign drugs. He argues: "Our English bodies are greatly divers from those of strange countries. If needs we will take unto us the practice of such strange medicines, it were to be desired that such medicines as be so perilous might be planted in our natural country, that through familiarity of our soil they might first grow into acquaintance with us before we entertain them into our hearts and chamber them with our vital spirits." Among rare and costly foreign remedies we find mention of rhubarb, then first coming into general repute from its home in the East. "T. B." mentions the existence of many excellent herb gardens in England, and especially in London, where a great variety of simples, many of foreign origin, could be obtained without recourse to the importer. The temptations to fraud and adulteration on the part of the dealer in these foreign drugs must have been irresistible in days when analysis was unknown and detection almost impossible, and "T. B." was no doubt on this account alone justified in his protest against any but home-grown articles.

The first book in English treating systematically of the study of simples was the "Great Herbal, 1526, which giveth perfect knowledge and understanding of all manner of herbs and their gracious virtues. Also it giveth perfect understanding of the book lately put forth by me, Peter Treveris, named the noble experience of the virtuous handwork of surgery." Of the surgical work thus referred to no trace remains, except in so far as the treatment of surgical cases enters into the plan of the herbal. A very accurately drawn skeleton forms the frontispiece of the volume, and the letterpress is adorned with a vast number of woodcuts, not only of herbs, but of stones, beasts, fishes, etc. It is pretty clear that Peter Treveris got his knowledge almost entirely from books, and was as neglectful of the evidence of his own eyes as any of his contemporaries. His authorities consist of the usual respectable band: "Avicenna, Pandectus, Constantinis, Wilhelmus, Platearius, Rabbi Moyses, Johannes Mesue, Haly Albertus, Bartholomew, and more other," including very plainly the elder Pliny. This magnificent book was evidently held in high esteem, to judge by the care with which it was issued, and its subsequent reprints. Further editions were put forth in 1529, and with numerous corrections and additions in 1539 and 1561. However useful it may have been held from a medical point of view, it can hardly have much advanced the knowledge of the real properties of herbs.

In 1580 Macer's Herbal, "practised by Doctor Linacro," was translated out of Latin into English. Dr. Linacer is a great name in the history of English medicine, and it is not a little surprising to find with what kind of scientific observations his name is here identified. The volume consists of a list of the commoner sorts of herbs in alphabetical order, setting forth first the description by which it may be recognised, and secondly, its medical properties. It is hard to say which are more amazing, the descriptions of the plants, or the qualities ascribed to them. Who could have been the writer's authority for the following charming invention, and why was he not immediately exposed as a manifest liar? "Astraton is an herb called Lunary; the leaves are zend blue, and they have the mark of the moon in the midst; this groweth in the new moon without leaves, and every day waxeth a new leaf, fifteen days, and after fifteen days he loseth a leaf as the moon waxeth and waneth. Eat for falling sickness, as the moon waneth and wear about the neck." Some of the properties are frankly magical. "Mugworte—artemesia—the vertue of this herb is if a man travelling bear this herb about him he should not be weary in his travel nor in his way. Also, where this herb is in any house, there shall no wicked spirit abide." St. John's Wort has also a like property of suffering no evil spirit to dwell in the house. Of rosemary the maiden is bidden "make a box of the wood and smell to it, and it shall preserve thy youth; put thereof in thy doors or in the house, and thou shalt be without danger of adder or venomous serpents." A certain plant called Lingua bovis, or Languebefe, drunk with hot water maketh a man to have a good mind and a good wit. A very ancient superstition is evidenced in the assertion concerning Lactuca leporica, or hare thistle, that if a hare eat of this herb in summer when he is mad he shall be whole. None of the prescriptions come much within the limits of practical medicine, and it is often puzzling to account for the qualities being ascribed to one plant rather than another. Elena campana, or horse helm, is described as a plant with a long stalk and yellow flower, with leaves like conifers, but whiter; of it we are told "if a man hath wagging teeth and eat thereof fasting it will fasten his teeth." Perhaps the most likely recommendation is that concerning annis, that "he unbindeth wicked winds and great humours," but it is not quite clear whether the "wicked winds" referred to are within or without.

Two or three other translations of this curious herbal are extant of a very early date, testifying to the great estimation in which it was held. It may be seen from the extracts given that these early herbals had no botanical value, and as they are mere reproductions of the ancients they give no real information about the knowledge of simples which actually prevailed at the period when they were compiled or translated. Any village herb doctor or prudent mother of a family could make herb teas of more practical value than those here devised.

But the way having once been pointed out it was not long before writers arose to carry on the work of classification on lines more nearly approaching those of modern scientific observation.

